# Prompting Decision Transformers for Zero-Shot Reach-Avoid Policies

**Published:** 2025-05-27

**Authors:** Kevin Li, Marinka Zitnik

**Affiliations:** Massachusetts Institute of Technology, Harvard Medical School; Harvard Medical School, Kempner Institute at Harvard University

## Abstract

Offline goal-conditioned reinforcement learning methods have shown promise for reach-avoid tasks, where an agent must reach a target state while avoiding undesirable regions of the state space. Existing approaches typically encode avoid-region information into an augmented state space and cost function, which prevents flexible, dynamic specification of novel avoid-region information at evaluation time. They also rely heavily on well-designed reward and cost functions, limiting scalability to complex or poorly structured environments. We introduce RADT, a decision transformer model for offline, reward-free, goal-conditioned, avoid region-conditioned RL. RADT encodes goals and avoid regions directly as prompt tokens, allowing any number of avoid regions of arbitrary size to be specified at evaluation time. Using only suboptimal offline trajectories from a random policy, RADT learns reach-avoid behavior through a novel combination of goal and avoid-region hindsight relabeling. We benchmark RADT against 3 existing offline goal-conditioned RL models across 11 tasks, environments, and experimental settings. RADT generalizes in a zero-shot manner to out-of-distribution avoid region sizes and counts, outperforming baselines that require retraining. In one such zero-shot setting, RADT achieves 35.7% improvement in normalized cost over the best retrained baseline while maintaining high goal-reaching success. We apply RADT to cell reprogramming in biology, where it reduces visits to undesirable intermediate gene expression states during trajectories to desired target states, despite stochastic transitions and discrete, structured state dynamics.

## 1 Introduction

Many high-risk sequential decision-making problems [26, 13, 1] are naturally framed as reach-avoid tasks [16, 37] [8], in which an agent must reach a designated goal state while avoiding undesirable regions of the state space. These problems arise in diverse domains, including robotics [13, 35, 3] (e.g., robotic arms reaching for targets while avoiding fragile objects), autonomous navigation [26, 1] (e.g., self-driving vehicles avoiding pedestrians), and biology [44, 25] (e.g., cell reprogramming strategies that aim to reach a therapeutic gene expression state without traversing tumorigenic intermediates). Despite domain-specific differences, these tasks share a common structure: they require balancing goal achievement with dynamic avoidance of specified hazards.

Solving reach-avoid problems is especially important in safety-critical environments where entering undesirable states can have irreversible consequences. These environments often preclude online exploration, making offline learning necessary [26]. Furthermore, in practical deployments, the specification of goals and avoid regions may change based on user preferences or environmental context. For instance, a robot assistant may need to adapt to new furniture layouts, or a therapeutic model may need to avoid different toxic intermediate states based on patient-specific risk factors. These settings require flexible and interpretable models that support zero-shot generalization to unseen goal and avoid specifications without retraining.

However, reach-avoid learning remains difficult. Most existing approaches rely on augmented state representations and carefully shaped cost functions to encode avoid behavior [3, 45, 51, 24, 23, 26]. This tight coupling of avoid-region semantics to model internals prevents flexible deployment and limits generalization. Reward-based formulations often struggle to represent multiple behavioral preferences simultaneously [1, 9, 19], especially when goal-reaching and avoidance conflict. Reward-free methods avoid these issues but lack a mechanism for dynamically conditioning behavior on new avoid constraints [12, 47, 17, 7]. Moreover, many offline approaches rely on expert demonstrations or near-optimal data to learn strong policies [32, 26, 3, 10, 21], which are often not available in safety-critical or high-dimensional tasks [11, 22, 29].

**Figure 1:**
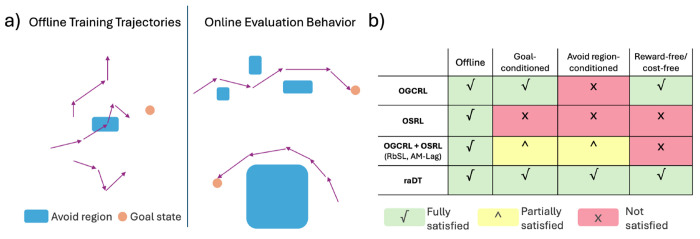
(a) An ideal reach-avoid model should learn to avoid arbitrarily specified regions of varying number and size at evaluation time, using only suboptimal, random-policy training data. (b) RADT is the only model that satisfies all criteria for an ideal reach-avoid learner (Section 2).

Several lines of work attempt to address parts of the reach-avoid problem. Offline goal-conditioned RL (OGCRL) approaches [7, 33, 12, 47, 27, 48, 20, 28, 17] learn policies from suboptimal data that can generalize to arbitrary goal states but cannot account for avoid constraints at test time. Offline safe RL (OSRL) approaches [51, 24, 45, 23] use cost functions and constraint optimization techniques like Lagrangian penalties [38] to learn constraint-satisfying policies, but they are often not truly goal-conditioned and avoid region-conditioned, and thus cannot generalize to arbitrarily specified goals and avoid regions within the state space at evaluation time. Recent offline goal-conditioned safe RL methods [3, 8] combine these ideas, but typically hardcode the number and size of avoid regions into the augmented state space and cost function designs, require retraining when these values change. These approaches fail to meet key criteria needed for flexible and safe reach-avoid learning: offline learning from suboptimal data, dynamic conditioning on arbitrary goals and avoid regions, and reward-free training (Figure 1).

### Present work.

We introduce RADT (Reach-Avoid Decision Transformer), a reward-free, offline RL model for goal-conditioned and avoid region-conditioned reach-avoid learning (Figure 1). RADT is a decision transformer that represents goals and avoid regions as prompt tokens. This formulation decouples the reach-avoid specification from the state representation and enables zero-shot generalization to arbitrary numbers and sizes of avoid regions. RADT learns policies entirely from random-policy trajectories using a novel combination of goal and avoid-region hindsight relabeling, with no need for reward or cost functions. Our main contributions include: ① A prompting framework for reach-avoid learning that encodes goals and multiple avoid regions as discrete prompt tokens, allowing flexible and interpretable conditioning of behavior at test time. ② A novel avoid-region hindsight relabeling strategy that allows the model to learn successful avoid behavior from suboptimal data. ③ A decision transformer model trained on random-policy data with no reward or cost functions, enabling reach-avoid learning in entirely offline, reward-free settings. ④ Benchmarking across robotics and biological domains evaluates generalization to out-of-distribution avoid-region sizes and counts. ⑤ Strong empirical results showing that RADT generalizes zero-shot to 8 unseen reach-avoid configurations, outperforming 3 existing methods retrained directly on those configurations.

## 2 Desirable Properties of Reach-Avoid RL Models

### Notation.

We first establish the notation used throughout this work. A trajectory τ of length T is a sequence of alternating states and actions: $\tau =\left(s_{1},a_{1},s_{2},a_{2},\ldots ,s_{T},a_{T}\right)$, where $s_{t}\in {\mathbb{R}}^{d_{s}}$ is the state at time t and $a_{t}\in {\mathbb{R}}^{d_{a}}$ is the action taken from $s_{t}$. The state and action spaces are denoted 𝒮 and 𝒜 with dimensions $d_{s}$ and $d_{a}$, respectively. All RL models in this work learn a deterministic policy π(a∣⋅) that selects the most preferred action given contextual inputs, typically including the current state $s_{t}$. If used, the reward function $r\left(s,a,s^{\prime }\right)$ and cost function cost$\left(s,a,s^{\prime }\right)$ return scalar values based on a transition tuple ($s,a,s^{\prime }$). When applicable, these values are included in the trajectory as: $\tau =\left(s_{1},a_{1},r_{1},c_{1},s_{2},a_{2},\ldots \right)$. In goal-conditioned settings, a goal g∈𝒮 is provided as an additional input, yielding conditional functions such as π(a∣⋅,g) or $r\left(s,a,s^{\prime },g\right)$. In avoid-region-conditioned settings with $n_{\text{avoid}}$ avoid regions $b_{j}:j\in \left\{1,2,\ldots ,n_{\text{avoid}}\right\}$, similar conditioning applies. We refer to the center of avoid region $b_{j}$ as its avoid centroid, denoted centroid($b_{j}$).

### Desirable Properties.

Reach-avoid problems introduce a dual behavioral objective: the agent must reach a desired target state while avoiding explicitly defined regions of the state space. Reach-avoid models that satisfy this behavioral objective under real-world deployment constraints need to achieve the following key properties.

### Property (1): Pre-collected offline datasets with no online fine-tuning.

The model must learn solely from offline datasets 𝒟 containing of pre-collected trajectories $\tau^{\left(i\right)}$, with no reliance on online fine-tuning. In safety-critical applications, online exploration may be infeasible, especially when entering avoid regions could cause irreversible harm or failure.

### Property (2): Suboptimality-tolerant learning.

The model must learn optimal or near-optimal policies from offline datasets that contain only suboptimal trajectories, i.e., those that do not reach the goal or that violate the avoid constraint. Specifically, it should support super-demonstration performance by learning from trajectories $\tau^{\left(i\right)}$ that: **(2.1)** fail to reach the target goal state g at rollout time, and/or **(2.2)** pass through avoid regions $b_{j}$ rather than successfully avoiding them.

### Property (3): Goal-conditioned generalization.

The model must generalize to any arbitrarily specified goal state g at evaluation time, without additional training. In real-world scenarios, such as autonomous navigation or therapeutic reprogramming, the target goal is often specified dynamically and cannot be hardcoded at training time.

### Property (4): Avoid region-conditioned generalization.

The model must be able to learn a policy that can avoid any dynamically specified avoid region(s) $b_{j}$ of the state space at evaluation time, without additional training/finetuning. This includes supporting changes in: **(4.1)** the number of avoid regions $n_{\text{avoid}}$, **(4.2)** their locations centroid($b_{j}$), and **(4.3)** their sizes (i.e., spatial extent of the state space around each centroid($b_{j}$) to avoid).

### Property (5): Reward-free learning.

The model must learn reach-avoid behavior without requiring a manually designed reward or cost function. Reward shaping is often brittle and requires expert domain knowledge [9, 18, 19, 1], especially when preferences over reaching and avoiding are difficult to encode or conflict. Instead, one should be possible to specify goals and avoid regions directly as inputs to the model.

While many prior approaches address subsets of these properties, none satisfy all five simultaneously (Figure 1b). We discuss these limitations in detail in Section 3.

## 3 Related Work

We review four key areas in reach-avoid learning: offline goal-conditioned RL, offline safe RL, offline goal-conditioned safe RL, and decision transformer-based models. Figure 1b summarizes which properties each class of methods satisfies. Additional discussion appears in Appendix E.

### Offline Goal-Conditioned RL.

Offline goal-conditioned RL (OGCRL) methods aim to learn policies that generalize to arbitrary goals specified at evaluation time, typically by conditioning on goal states and applying techniques such as hindsight goal relabeling [2]. These methods fall into two broad categories. **(1) Reward-based OGCRL** methods [48, 20, 28] optimize a policy π(a∣s,g) to maximize a goal-conditioned reward function $r\left(s,a,s^{\prime },g\right)$. While this framework supports goal generalization (Property (3)), it does not satisfy Property (5), as it requires designing reward signals. In practice, reward functions that capture both goal-reaching and avoid behavior are difficult to construct [18, 19, 9], making these approaches brittle in reach-avoid settings. **(2) Reward-free OGCRL** methods [7, 33, 12, 47, 27, 17] avoid reward functions by learning from hindsight-relabeled trajectories using supervised learning. These methods satisfy Property (5) but do not support avoid-region conditioning (Property (4)), as they cannot incorporate constraints beyond the goal.

### Offline Safe RL.

Offline safe RL (OSRL) methods [51, 24, 45, 23] are designed for safety-critical tasks, learning policies that satisfy constraints specified via cost functions. A typical approach defines a cost function cost$\left(s,a,s^{\prime }\right)$ and learns a policy that maximizes reward return $\sum_{t}r_{t}$ subject to a cost return constraint $\sum_{t}c_{t}<k$, often via Lagrangian relaxation [38]. These methods can enforce avoid behavior, but they are not generally goal-conditioned (Property (3)), and do not support dynamic conditioning on varying avoid-region configurations (Property 4). In addition, these methods fail Property (5) due to their reliance on handcrafted reward and cost functions.

### Offline Goal-Conditioned Safe RL.

This hybrid category combines elements of OGCRL and OSRL and comes closest to satisfying the full set of reach-avoid properties. Representative methods include Recovery-based Supervised Learning (RbSL) and Actionable Models with Lagrangian Constraints (AM-Lag) [3, 4, 38]. These models construct an augmented state space ${𝒮}^{+}\;\subseteq \;{\mathbb{R}}^{d_{s}+d_{s}+n_{\text{avoid}}\cdot d_{s}}$ containing agent state, goal g, and avoid centroids centroid$\left(b_{j}\right)$, and learn policies with goal-conditioned and avoid-conditioned objectives. This design satisfies Properties (3) and (4.2), enabling generalization to arbitrary g and avoid-region locations. However, these methods do not satisfy Property (4.1), as the number of avoid regions $n_{\text{avoid}}$ is fixed in the state space dimension, requiring retraining to accommodate more regions. They also fail to support Property (4.3), as the spatial extent of avoid regions is encoded only in the cost function, requiring redefinition and retraining when region size changes. Furthermore, although these models could in principle satisfy Property (2.2) (learning from trajectories that violate avoid regions), the training data in [3] includes physical, impassable obstacles, meaning no training trajectories actually pass through avoid regions (Figure 3b). This means training data is not entirely suboptimal. See Appendix C.1 for details.

### Decision Transformers.

Decision Transformer (DT) models [5, 17, 50, 43, 42] represent a class of offline RL approaches that frame policy learning as sequence modeling. DTs use causal transformer architectures that take as input a trajectory τ and autoregressively predict actions, $\pi \left(a_{t}|\tau_{1:t-1},s_{t}\right)$. Prompting has recently been introduced as a mechanism to extend DTs to goal-conditioned settings [46, 49]. We build our method off of MGPO [49], which introduces prompt-based conditioning on arbitrary goals, enabling zero-shot generalization across goal states, but not avoid regions.

## 4 Reach-Avoid Decision Transformer (RADT)

**Figure 2:**
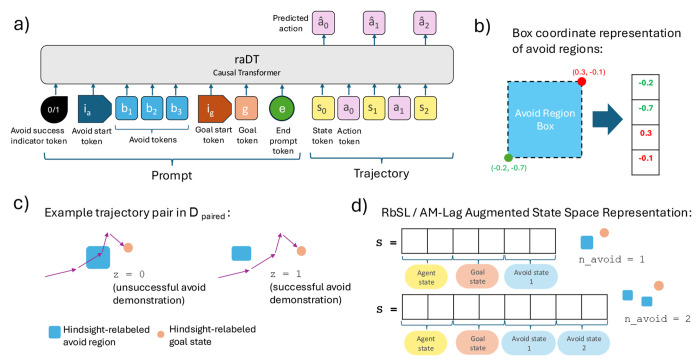
(a) RADT receives goal states and avoid regions as prompt inputs. (b) Avoid regions are defined as boxes in the state space and encoded as vectors of bounding box corner coordinates. (c) For each offline trajectory, we generate two versions: one that violates a sampled avoid region and one that avoids it. Both are labeled with an avoid success token *z*. (d) Prior models encode avoid regions via augmented state vectors, which grow with the number of avoid regions, preventing zero-shot generalization to unseen avoid counts.

In this section, we describe the main components of our method, RADT (Figure 2). Similar to MGPO, our method is based on a causal Transformer architecture and utilizes prompts to specify goal states (satisfying Property (3)). However, unlike MGPO, RADT additionally allows for the specification of avoid regions in the prompt (satisfying Property (4)) and does not require reward-driven online prompt optimization (satisfying Properties (1), (2), and (5)).

### 4.1 Prompt Tokens and Avoid Region Representation

RADT takes in a prompt p to be presented to the Transformer model before a trajectory τ. RADT’s autoregressive prediction of the next action during rollout is thus additionally conditioned on this prompt: $\pi \left(a_{t}|p,\tau_{0:t-1},s_{t}\right)$. The prompt is structured as follows (Figure 2a):

$$p=\left(z,i_{b},b_{1},b_{2},\ldots ,b_{n_{\text{avoid}}},i_{g},g,e\right)$$

Note that there are *six different types* of tokens present in the prompt: ① **The avoid success indicator token,** z:z∈{0, 1}, indicates whether the trajectory τ following the prompt successfully circumvents the avoid state:

$$z=\left\{\begin{array}{rr} 1 & \text{if all states in}\;\tau \;\text{exist outside of all avoid boxes}\;b_{j}:j\in \left\{1,2,\ldots ,n_{\text{avoid}}\right\}\\ 0 & \text{otherwise} \end{array}\right.$$

This allows us to train the model on both trajectories that demonstrate successful and unsuccessful avoid behaviors; this is important for the model to explicitly learn what *not* to do (see Section 4.2). During evaluation time, we will always condition on z=1 to achieve optimal avoid behavior. ② **The avoid start token**, $i_{b}$, explicitly indicates to the model that the upcoming tokens represent undesirable avoid regions. This is to clearly distinguish the avoid tokens from the avoid success indicator token. ③ **The avoid tokens**, $b_{j}\in {\mathbb{R}}^{2*d_{s}}\;:\;j\in \left\{1,2,\ldots ,n_{\text{avoid}}\right\}$, represents the $n_{\text{avoid}}$ avoid regions we would like to circumvent, represented as *box coordinates* (Fig 2b). The box coordinate vector of an avoid region $b_{j}$ represents a “box” in the state space to be avoided. It is defined such that the first $d_{s}$ entries represent the lower bounds of each of the state space dimensions (the “lower left corner” of the box in a 3D analogy) and the second $d_{s}$ entries represent the upper bounds of each of the state space dimensions (the “upper right corner”):

$$b_{j}=\underset{\text{lower bounds}}{\underbrace{\left[l_{1},l_{2},\ldots ,l_{d_{s}}\right.}},\overset{\text{upper bounds}}{\overbrace{u_{1},u_{2}\ldots ,u_{d_{s}}}}]$$

The policy should avoid guiding the agent into the region of the state space bounded by this box. Because we represent avoid regions as boxes in the state space, we will also use the term “avoid box” to refer to avoid regions. Since prompts can consist of any arbitrary number of avoid tokens $b_{j}$ and the avoid tokens can be boxes of any arbitrary size at evaluation time, this satisfies Properties (4.1), (4.2), and (4.3). ④ **The goal start token**, $i_{g}$, explicitly indicates to the model that the next token to be provided represents a desirable goal token. This is to clearly distinguish the goal token in the prompt from the avoid tokens. ⑤ **The goal token**, $g\in {\mathbb{R}}^{d_{s}}$, represents the state we would like to achieve. This can be set to any state in the state space at evaluation time (satisfying Property (3)). ⑥ **The prompt end token**, e, explicitly marks the end of the prompt. This indicates to the model that the next token marks the beginning of the main input sequence, τ.

See Appendix B.1 for details regarding how these different token types are embedded. Since we use prompts to specify the goal and avoid region information, we do not need to work with an augmented state space ${𝒮}^{+}$, unlike RbSL and AM-Lag (see 3), providing us with greater zero-shot flexibility.

### 4.2 Avoid Region Relabeling and Training Data

We specifically consider the scenario in which the training dataset 𝒟 contains $\tau^{\left(i\right)}$ that are generated from a purely random policy (satisfying Properties (1) and (2)). For each training trajectory $\tau^{\left(i\right)}\in 𝒟$, we relabel the last state $s_{T}$ as the goal state g in hindsight. Additionally, we can also relabel *avoid regions* in hindsight, a novel strategy that gets rid of the need to use a cost function to learn desirable avoid behavior. The intuition for hindsight avoid region relabeling is similar to goal relabeling; it does not matter whether the policy that collected the training trajectory was *actually trying* to circumvent the hindsight-relabeled avoid region; we can still treat it as a region that was “meant” to be avoided, as the trajectory demonstrates how to *not* pass through that region.

For each $\tau^{\left(i\right)}\in 𝒟$, we carry out hindsight avoid relabeling in two passes. In the initial pass, we randomly sample avoid boxes $b_{j}\;:\;j\in \left\{1,2,\ldots ,n_{\text{avoid}}\right\}$ of random sizes in the state space 𝒮 and check whether any $s_{t}\in \tau^{\left(i\right)}$ violate any $b_{j}:j\in \left\{1,2,\ldots ,n_{\text{avoid}}\right\}$. If there are no violations, then the avoid success indicator for $\tau^{\left(i\right)}$ is set to $z^{\left(i\right)}=1$, otherwise it is set to $z^{\left(i\right)}=0$. In the second pass, we create a copy of the dataset, ${𝒟}_{\text{copy}}$, and go through the same process above with the trajectories ${\tau_{\text{copy}}}^{\left(i\right)}\in {𝒟}_{\text{copy}}$. This time, however, for a trajectory ${\tau_{\text{copy}}}^{\left(i\right)}$, we keep re-sampling avoid boxes until the avoid success token ${z_{\text{copy}}}^{\left(i\right)}$ is the *opposite* of $z^{\left(i\right)}$ for the corresponding $\tau^{\left(i\right)}$ in the original 𝒟. Refer to Appendix A.2 for more detailed overview.

Combining the datasets 𝒟 and ${𝒟}_{\text{copy}}$ into ${𝒟}_{\text{paired}}$, we now have a *pair* of trajectories $\left(\tau_{\text{orig}}^{\left(i\right)},\tau_{\text{copy}}^{\left(i\right)}\right)$ for each $\tau^{\left(i\right)}$ in the original dataset, where one of $\left(\tau_{\text{orig}}^{\left(i\right)},\tau_{\text{copy}}^{\left(i\right)}\right)$ demonstrates successful avoid behavior and the other demonstrates unsuccessful avoid behavior. The intent is to isolate the concept of avoid success vs. failure to the model from differences in the trajectories themselves. This way, the model can more clearly learn what the avoid success token z represents conceptually (Figure 2c). Like other DT-based models, the training objective is a causal language modeling objective (Appendix B.2).

## 5 Experiments

We evaluate RADT in three reach-avoid environments: FetchReachObstacle, MazeObstacle, and CardiogenesisCellReprogramming. The first two are adapted from Gymnasium Robotics tasks, FetchReach and U-Maze [6], with added avoid regions. In these environments, we compare RADT against two offline reach-avoid baselines: RbSL [3] and AM-Lag [3, 4, 38], as well as Weighted Goal-Conditioned Supervised Learning (WGCSL) [47], a strong offline goal-conditioned method that does not explicitly account for avoid regions. To evaluate the generality of RADT, we also test it in a biological setting: generating safe gene expression trajectories for cell reprogramming. Additional details on task setup and design choices are provided in Appendix C.

### 5.1 Fetch Reach Environment and Generalization to Varying Avoid Region Sizes

#### Environment.

**Figure 3:**
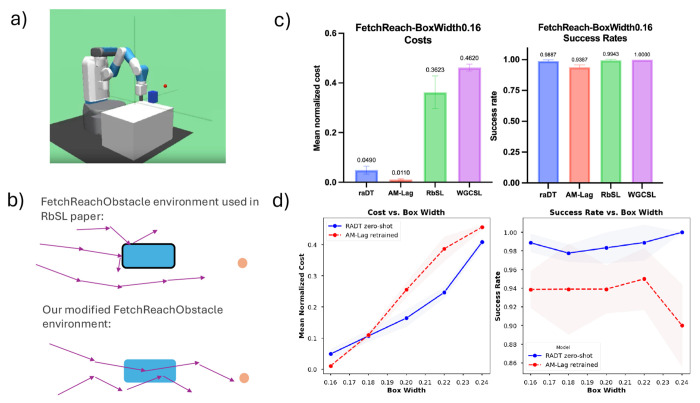
(a) Visualization of the FetchReachObstacle environment. The red point is the goal; the blue box is the avoid region. (b) Unlike prior setups, the robot arm can pass through avoid boxes, allowing training data to include violations. (c) RADT and AM-Lag achieve state-of-the-art reach-avoid performance on in-distribution box sizes, measured by MNC and SR. (d) RADT generalizes zero-shot to out-of-distribution avoid box sizes, matching or surpassing the best baseline (AM-Lag), which needs to be *retrained* on every new avoid box size.

The FetchReachObstacle environment requires a robotic arm to reach a goal location while avoiding a randomly positioned box, treated as the avoid region under our formulation (Figure 3a). The positions of the end-effector, goal, and avoid box are randomly sampled each episode. Unlike prior work such as [3], the avoid box here is not physical; the robot can pass through it, allowing us to isolate avoid-region reasoning without hard dynamics constraints (Figure 3b).

#### Data.

We collect 2 million timesteps of random-policy trajectories. Avoid-region hindsight relabeling is applied as described in Section 4.2, using a contour-based sampling strategy to generate diverse avoid box placements (Appendix A.2).

#### Evaluation Metrics.

While RADT is trained without a cost function, we define one for evaluation: $cost\left(s_{t},a_{t},s_{t+1}\right)=1$ if $s_{t+1}$ is inside an avoid box; otherwise 0. We evaluate models using the normalized cost return (MNC), computed as the average per-step cost: $MNC(\tau )=\frac{1}{\left|\tau \right|}\sum_{\left(s,a,s^{\prime }\right)\in \tau }\;cost\left(s,a,s^{\prime }\right)$. We also report the goal-reaching success rate (SR), defined as the proportion of episodes that reach the goal. Evaluation is performed over 60 episodes.

#### Setup.

We train all models using environments with a fixed avoid box width (or, in the case of RADT, a max hindsight relabeled box width) of 0.16. RbSL, AM-Lag, and WGCSL are trained using the above cost function. We then evaluate all models in environments with the same 0.16 avoid box width (in-distribution). To assess generalization, we evaluate RADT zero-shot on avoid boxes with widths ranging from 0.16 to 0.24 (1.5× larger). In contrast, baseline models do not support zero-shot generalization to new avoid box sizes and must be retrained on separate offline datasets generated for each new size (Sections 3 and 4.1, and Appendix C.1). This creates a disadvantageous setting for RADT, which is evaluated without retraining, while baselines are retrained for each test condition.

#### Results.

Results are shown in Table 1. For the in-distribution case (box width 0.16), RADT performs comparably to AM-Lag in MNC and better than RbSL and WGCSL. For SR, RADT outperforms AM-Lag and matches or exceeds the other baselines (Figure 3c). This confirms that both RADT and AM-Lag are competitive for reach-avoid learning, with RADT slightly favoring success rate and AM-Lag slightly favoring cost minimization. In the out-of-distribution (OOD) setting, where avoid box sizes exceed those seen during training, RADT continues to perform strongly despite being evaluated zero-shot. Across all OOD widths, RADT matches or exceeds the MNC of retrained AM-Lag models and substantially outperforms retrained RbSL and WGCSL models. It also maintains comparable SR to the retrained baselines (Figure 3d). These results show that RADT generalizes to unseen avoid-region sizes, even outperforming methods that are *retrained* for each evaluation setting.

### 5.2 Maze Environment and Generalization to Varying Numbers of Avoid Regions

#### Environment.

The MazeObstacle environment requires a point agent to navigate through a U-shaped maze to reach a randomly sampled goal location while avoiding randomly placed circular obstacles. As in FetchReachObstacle, these obstacles are soft constraints that the agent may pass through, and we refer to them as “avoid regions.” Unlike in FetchReachObstacle, the maze itself imposes hard constraints, introducing impassable regions of the state space. This tests RADT’s ability to reason over both user-specified avoid regions and inherent environmental constraints.

#### Data and Evaluation.

Data and Evaluation pipeline is, for the most part, the same as in FetchReachObstacle, except using a simpler sampling method for avoid relabeling. See Appendix A.2 for details.

#### Setup.

We begin by training all models using data generated in environments with a single avoid region, and evaluate in matching single-avoid-region settings (in-distribution). To evaluate generalization, we then train and evaluate all models in environments with three avoid regions, followed by testing in environments with 4-7 avoid regions to assess zero-shot out-of-distribution (OOD) performance. For each new number of avoid regions, baseline models (RbSL, AM-Lag, WGCSL) are retrained with an appropriately expanded state space (Figure 2d; see Sections 3 and 4.1). In contrast, RADT is evaluated zero-shot without any retraining. This design intentionally favors the baselines, as they are tailored to each test setting, whereas RADT is held fixed across all configurations.

#### Results.

Results are shown in Table 2. Across all settings (both in-distribution and OOD), RADT achieves the lowest MNC, consistently outperforming all baselines. It also outperforms AM-Lag and RbSL on SR, and performs comparably or better than WGCSL. Importantly, for all evaluations with more than three avoid regions, RADT is deployed zero-shot, while the baselines are retrained. Despite this disadvantage, RADT matches or exceeds their performance, demonstrating its ability to generalize to OOD numbers of avoid regions without retraining.

### 5.3 Applications in Cellular Biology: Zero-Shot Avoidance in Stochastic Reprogramming

**Figure 5:**
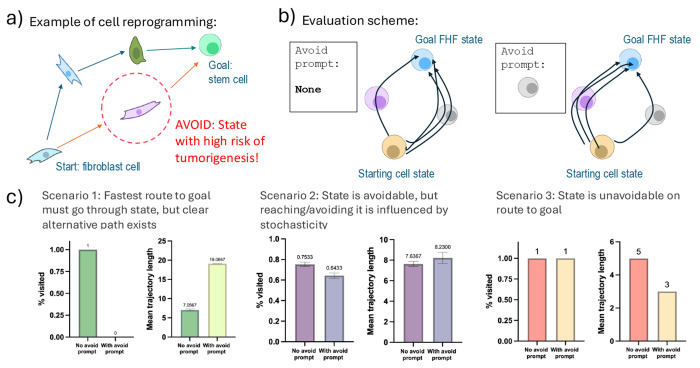
(a) Cell reprogramming involves sequential gene perturbations to reach a target expression state while avoiding unsafe intermediate states. (b) Evaluation pipeline: RADT is first run without an avoid token. The most frequently visited intermediate state (e.g., gray cell state) is then added as an avoid token, and RADT is re-evaluated. Ideally, the new trajectories will go through the gray cell state less often. (c) RADT reduces visitation frequency to specified avoid states and, when avoidance is infeasible, minimizes time spent in those states. Error bars show ±1 standard deviation. Some illustrations adapted from NIAID NIH BIOART (Appendix G).

We apply RADT to a biomedical reach-avoid problem: cell reprogramming. The goal is to transition a cell from one gene expression state to another using sequences of genetic perturbations. This technique underpins regenerative medicine [41, 40], stem cell therapy [39, 31, 14], and anti-aging strategies [30, 34]. However, intermediate gene expression states encountered during reprogramming may carry elevated risks, e.g. tumorigenesis [44, 25]. Safe reprogramming fits the reach-avoid formulation: reach a target state while avoiding undesirable intermediate states (Figure 5a).

#### Environment.

We introduce CardiogenesisCellReprogramming, an environment based on a well-established Boolean network model of gene expression dynamics during mouse cardiogenesis [15, 36]. The model comprises 15 genes, each represented by a binary variable indicating expression (1) or non-expression (0), yielding a discrete state space of size 2^15^. Actions correspond to genetic perturbations that flip the expression value of a single gene. After perturbation, the Boolean network asynchronously updates the system to a new gene expression state based on its internal logic. Unlike the Gymnasium Robotics environments, this domain features: (1) a fully discrete and combinatorial state-action space, (2) high-dimensional interdependencies between state variables due to Boolean logic, and (3) stochastic transitions. See Appendix C.2 for full environment specifications.

#### Data.

We generate 60,000 timesteps of training data by executing a random walk through the environment. Our hindsight avoid-region relabeling approach (Section 4.2) is directly applicable to this discrete setting with minimal modification (Appendix C.2).

#### Setup.

We conduct a case study where the reprogramming goal is to reach the first heart field (FHF) state, a critical attractor in cardiac development [15, 36]. We train RADT on the offline data, then evaluate its ability to reach the FHF state starting from a distinct attractor state (Appendix C.2). In the first condition, we run 200 evaluation episodes with no avoid region specified. From these, we identify the most frequently visited intermediate state, defined by: *percent visited*, where Percent Visited(*s*) = {# of trajectories that visit *s* at some point}/{# of total trajectories}. We then run a second set of 200 episodes, this time providing the most visited intermediate state as an avoid token in the prompt. We evaluate whether RADT is able to discover alternative reprogramming paths that avoid this state and compare both visitation rates and trajectory lengths. This setup is illustrated in Figure 5b. See Appendix C.2 for details.

#### Results.

All evaluation trajectories successfully reach the FHF goal state, so we report only intermediate state visitation and trajectory lengths. Figure 5c summarizes results. We observe three distinct behavioral patterns, depending on the initial state. **Scenario 1.** The most visited intermediate state (state A) is encountered in every trajectory when no avoid token is used. When A is included as an avoid token, RADT avoids it entirely and instead follows longer trajectories that bypass A. The trajectory length increases significantly, confirming that RADT successfully discovers an alternative path that trades off efficiency for safety. **Scenario 2.** Another state (state B) is frequently visited but not on all trajectories, even without avoid conditioning. When B is specified as an avoid token, its visitation rate decreases, but does not drop to zero. This suggests that while B is not essential to reach the goal, it lies along many probable trajectories due to stochastic dynamics. RADT still reduces visitation frequency in this noisy setting. **Scenario 3.** The most visited state (state C) is present in all trajectories regardless of prompt. When C is added as an avoid token, RADT cannot avoid passing through it, indicating that it is structurally unavoidable from the given initial state. However, RADT reduces the number of steps spent in C, shortening the portion of the trajectory that includes it. This suggests that even when avoidance is infeasible, the model learns to minimize time spent in unsafe states. These results highlight that RADT supports reach-avoid planning in discrete, structured, and stochastic domains, and also exhibits flexible avoidance strategies, including temporal minimization of contact with undesirable states when full avoidance is not possible. Full results and trajectory visualizations are in Appendix C.2.

## 6 Conclusion

We introduce RADT, a prompting-based offline reinforcement learning model for reach-avoid tasks that satisfies all key desiderata for flexible, reward-free learning. RADT generalizes zero-shot to unseen avoid region configurations, varying both number and size without retraining. It is trained entirely on suboptimal data, without expert demonstrations or rewards, and achieves competitive or superior performance to state-of-the-art methods re-trained for each evaluation setting. RADT is domain-agnostic, demonstrating strong results in both robotics and cell reprogramming, including reduced visitation of unsafe gene expression states. We attribute RADT ’s versatility to its interpretable prompt-based design, which encodes reach and avoid specifications as input tokens, and its fully data-driven learning procedure that does not rely on reward shaping or constraint tuning. These properties enable RADT to serve as a general-purpose framework for safe sequential decision-making under dynamically shifting constraints.

### Limitations.

The flexibility and zero-shot capabilities of RADT are balanced out by the fact that it takes a lot longer and a lot more computational resources to train compared to the baselines models, having many more parameters (as it is based on a GPT-2 architecture). Additionally, the current iteration of RADT can only handle avoid boxes, and while we have been using conservative box sizes for avoid regions that are not inherently rectangular, applications that require tight boundaries for avoid regions of more complex shapes will be difficult for our model.

## Figures and Tables

**Figure 4:**
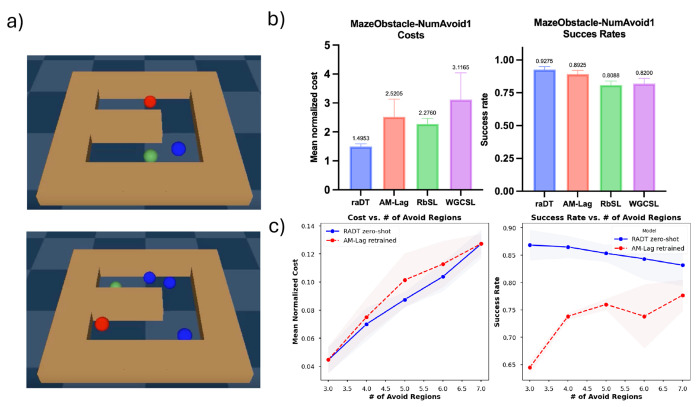
(a) Visualization of the MazeObstacle environment, with red goal, blue avoid regions, and green agent. (b) RADT outperforms all baselines on MNC and SR in the in-distribution single-avoid setting. (c) RADT generalizes zero-shot to out-of-distribution numbers of avoid regions, matching the best retrained baseline (AM-Lag) in MNC and surpassing it in SR. Note that AM-Lag is *retrained* on every new number of avoid regions (i.e., non zero-shot). Error bars show ±1 standard deviation.

**Table 1:** Results for FetchReachObstacle with varying box sizes (avg. over 3 seeds).

Box Width	RADT^#^		AM-Lag		RbSL		WGCSL	
	MNC	SR	MNC	SR	MNC	SR	MNC	SR
0.16	**0.049**_±0.016_	**0.989**_±0.01_	**0.011**_±0.003_	0.95_±0.029_	0.362_±0.066_	**0.994**_±0.01_	0.462_±0.0148_	**1.0**_±0.0_
0.18	**0.107**_±0.018_	0.978_±0.009_	**0.11**_±0.01_	0.95_±0.033_	0.484_±0.016_	**1.0**_±0_	0.513_±0.048_	**0.994**_±0.01_
0.20	**0.164**_±0.027_	**0.983**_±0.017_	0.255_±0.023_	0.955_±0.025_	0.571_±0.013_	**1.0**_±0.0_	0.588_±0.066_	**1.0**_±0.0_
0.22	**0.247**_±0.018_	**0.989**_±0.019_	0.387_±0.039_	0.944_±0.02_	0.64_±0.053_	**0.99**_±0.01_	0.699_±0.019_	**0.994**_±0.01_
0.24	**0.409**_±0.012_	**1.0**_±0.0_	**0.457**_±0.008_	0.964_±0.017_	0.701_±0.019_	**1.0**_±0_	0.729_±0.038_	**0.989**_±0.01_

# = Model capable of zero-shot generalization.

Results highlighted in blue are zero-shot results.

**Table 2:** Results for MazeObstacle with varying number of avoid states (avg. over 3 seeds).

# Avoid	RADT^#^		AM-Lag		RbSL		WGCSL	
	MNC (1e-2)	SR	MNC (1e-2)	SR	MNC (1e-2)	SR	MNC (1e-2)	SR
1	**1.495**_±0.096_	**.928**_±0.022_	2.521_±0.613_	0.893_±0.029_	2.276_±0.193_	0.809_±0.031_	3.117_±0.922_	0.82_±0.041_

3	**4.455**_±0.895_	**0.868**_±0.028_	**4.47**_±0.94_	0.645_±0.01_	5.857_±0.754_	0.175_±0.054_	6.92_±0.404_	**0.842**_±0.033_
4	**7.006**_±1.156_	**0.865**_±0.02_	7.511_±1.342_	0.738_±0.006_	7.648_±0.357_	0.768_±0.043_	9.62_±1.701_	**0.852**_±0.043_
5	**8.75**_±0.531_	**0.853**_±0.015_	10.14_±1.9_	0.76_±0.01_	9.622_±0.531_	0.053_±0.012_	10.28_±0.503_	**0.807**_±0.033_
6	**10.38**_±1.015_	**0.843**_±0.038_	11.278_±1.629_	0.738_±0.058_	11.35_±1.604_	0.755_±0.065_	12.364_±1.414_	**0.9**_±0.017_
7	**12.7**_±1.0_	**0.832**_±0.038_	**12.72**_±0.702_	0.777_±0.028_	24.17_±3.65_	0.002_±0.003_	14.48_±1.439_	**0.825**_±0.018_

# = Model capable of zero-shot generalization.

Results highlighted in blue are zero-shot results.

**Figure 6:**
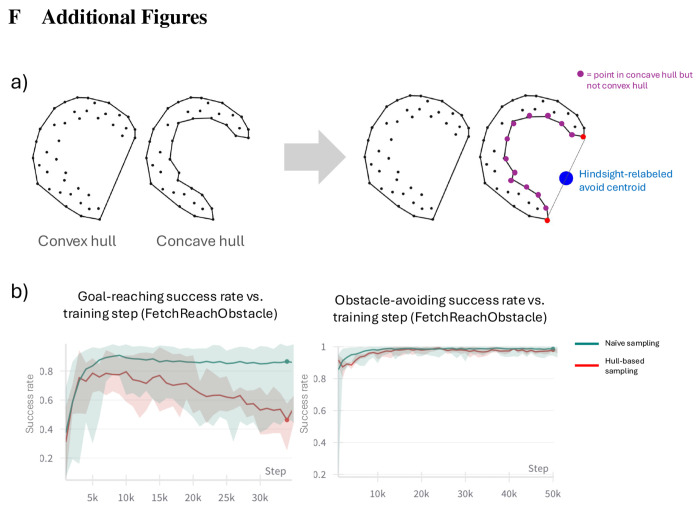
a) 2D depiction of the contour-based centroid sampling strategy for avoiding region relabeling. The convex and concave hulls are calculated for the set of data points in the state space for a training trajectory. Points that are part of the concave hull but not the convex hull (depicted here in purple) comprise concave portions of the trajectory that can be viewed as “wrapping around” some unknown avoid centroid. To generate an avoid centroid that fits this intuitive interpretation in hindsight, we locate the two points bordering this concave region (depicted here in red) and sample a point in between them. The resulting trajectory of data points looks like it’s “trying” to avoid the blue hindsight-relabeled avoid centroid by wrapping around it. Figure built upon an illustration from [60]. b) Using the contour-based strategy for sampling avoids centroids results in a higher maximum SR for the FetchReachObstacle environment and notably mitigates/delays overfitting.

**Figure 7:**
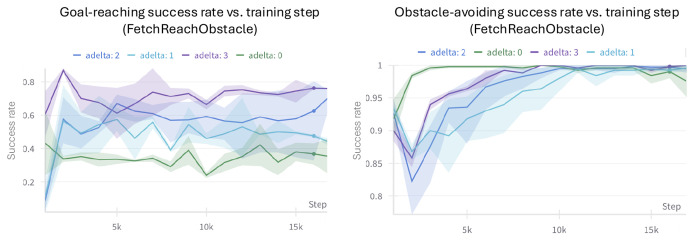
The maximum goal-reaching success rate for RADT trained on the FetchReachObstacle task improves with increasing the attention boosting bias adelta to the prompt tokens. The maximum obstacle-avoiding success rate, on the other hand, seems to be unaffected by the value of adelta.

**Figure 8:**
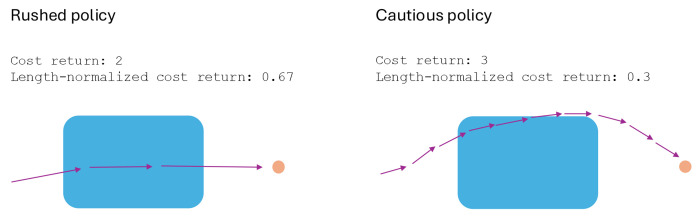
A policy that rushes directly through the avoid region to get to the goal as quickly as possible may earn a low absolute cost return but a high length-normalized cost return. On the other hand, a slower, more cautious policy that takes more timesteps to reach the goal but attempts to circumvent the avoid region may accumulate a similar absolute cost return, but a lower length-normalized cost return.

**Figure 9:**
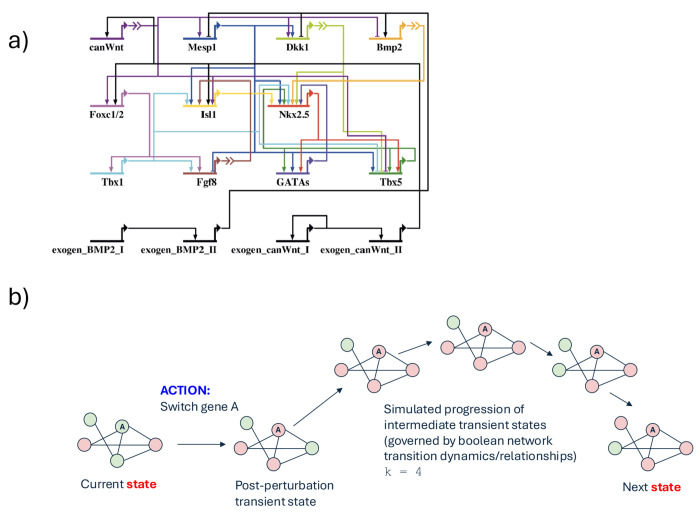
a) A diagram representing the 15-gene boolean network model for mouse cardiogenesis. b) A depiction of one state-action-state transition simulated in the Cardiogenesis environment, where k = 4.

## Broader Impacts

Foundational methods for learning reach-avoid policies, like RADT, have strong potential in improving the performance of technologies in many application domains, such as general robotics, autonomous vehicles, and bioengineering. While we believe the advancement of these downstream domains can greatly benefit society, we do acknowledge that such technologies can also be used maliciously. RADT and any derivatives should never be used to create autonomous agents with harmful goals (e.g., self-navigating vehicles that maliciously target individuals) or to induce harmful biological states (e.g., programming cells to pathological cell states). RADT is designed with the intent of enabling the development of *safer* sequential decision making agents that can improve the convenience and health of individuals in our society.

## A Data Prep

### A.1 Datasets

The download link for the preprocessed datasets, as well as the setup and preprocessing code used to generate these datasets, will be released with the code repository for the project. See Appendix D.

### A.2 Avoid Relabeling Details

#### A.2.1 Avoid Relabeling Two-Pass Algorithm

The two-pass hindsight avoid region relabeling method introduced in Section 4.2 is described in more detail in Algorithm 1 (first pass) and Algorithm 2 (second pass).

**Algorithm 1:** Hindsight Avoid Region Relabeling: Initial Pass

**Require:** state space 𝒮, offline training dataset 𝒟, maximum avoid box width $w_{\max}$, number of avoid states $n_{\text{avoid}}$
1: **for** training trajectory $\tau^{\left(i\right)}$ in 𝒟 **do**
2: Initialize avoid regions list ${\text{b\_list}}^{\left(i\right)}\leftarrow []$
3: **for** *j* in 1, 2, …, *n*_avoid_ **do**
4: Randomly initialize avoid centroid $x_{j}\in 𝒮$
5: Randomly choose avoid box width $w\in \left[0,w_{\max}\right]$
6: Avoid box $b_{j}\leftarrow \text{concatenate}(x_{j}-\frac{w}{2},x_{j}+\frac{w}{2})$ ▷ box of width *w* centered around $x_{j}$
7: Append **b**_*j*_ to ${\text{b\_list}}^{\left(i\right)}$
8: **end for**
9: **if** All none of the states in $\tau^{\left(i\right)}$ are in any of the avoid boxes in ${\text{b\_list}}^{\left(i\right)}$ **then**
10: Avoid success $z^{\left(i\right)}\leftarrow 1$
11: **else**
12: Avoid success $z^{\left(i\right)}\leftarrow 0$
13: **end if**
14: Add $z^{\left(i\right)}$ and ${\text{b\_list}}^{\left(i\right)}$ to the corresponding training prompt $p^{\left(i\right)}$ for $\tau^{\left(i\right)}$
15: **end for**

**Algorithm 2:** Hindsight Avoid Region Relabeling: Second Pass

**Require:** state space 𝒮, copy of the original offline training dataset ${𝒟}_{\text{copy}}$, maximum avoid box width $w_{\max},$, number of avoid states $n_{\mathrm{avoid}}$
1: **for** training trajectory ${\tau_{\text{copy}}}^{\left(i\right)}$ in ${𝒟}_{\text{copy}}$ **do**
2: ${z_{\text{copy}}}^{\left(i\right)}\leftarrow z^{\left(i\right)}$
3: **while** ${z_{\text{copy}}}^{\left(i\right)}=z^{\left(i\right)}$ **do**
4: Initialize avoid regions list ${\text{b\_list}}^{\left(i\right)}\leftarrow []$
5: **for** *j* in 1, 2, …, *n*_avoid_ **do**
6: Randomly initialize avoid centroid $x_{j}\in 𝒮$
7: Randomly choose avoid box width $w\in \left[0,w_{\max}\right]$
8: Avoid box $b_{j}\leftarrow \text{concatenate}(x_{j}-\frac{w}{2},x_{j}+\frac{w}{2})$ ▷ box of width *w* centered around **x**_*j*_
9: Append **b**_*j*_ to ${\text{b\_list}}^{\left(i\right)}$
10: **end for**
11: **if** All none of the states in $\tau^{\left(i\right)}$ are in any of the avoid boxes in ${\text{b\_list}}^{\left(i\right)}$ **then**
12: Avoid success ${z_{\text{copy}}}^{\left(i\right)}\leftarrow 1$
13: **else**
14: Avoid successs ${z_{\text{copy}}}^{\left(i\right)}\leftarrow 0$
15: **end if**
16: **end while**
17: Add ${z_{\text{copy}}}^{\left(i\right)}$ and ${\text{b\_list}}^{\left(i\right)}$ to the corresponding training prompt ${p_{\text{copy}}}^{\left(i\right)}$ for ${\tau_{\text{copy}}}^{\left(i\right)}$
18: **end for**

#### A.2.2 More Sophisticated Avoid Centroid Sampling Strategies

The sampling of avoid centroids in Line 4 of Algorithm 1 can be done using naive uniform sampling over the state space (the default) or more sophisticated methods.

##### Contour-Based Sampling.

One such sophisticated method we use samples avoid centroids that fit into the nooks of the broad contour of a training trajectory τ (Figure 6a). This is the sampling method used for the FetchReachObstacle environment (a very open environment with no inherent restricted areas of the state space). We can acquire an outline of the general *contour* of a τ by calculating a concave hull of the data points $s_{t}\in \tau$ in the state space 𝒮 using the algorithm presented in [56] as implemented the concave_hull library [59]. We denote the set of states in τ that belong to the convex hull as ${𝒮}_{\text{convex}}$. We can then find “nooks” (concave portions) of the trajectory by calculating the convex hull using the algorithm presented in [53] as implemented the concave_hull library, and then find the points that are part of the concave hull but not the convex hull; these points outline the concave nooks of the contour and we denote the set of these states as ${𝒮}_{\text{nook}}$. For each nook in trajectory τ, we then find the two convex hull points in ${𝒮}_{\text{convex}}$ bordering the set ${𝒮}_{\text{nook}}$ and sample a point between them as the avoid centroid. As a result, the trajectory of states *appears* to be “attempting” to avoid the hindsight-relabeled avoid centroid by wrapping around it (Figure 6a). Although the training trajectory was not trying to avoid this state, this does not matter because the resulting hindsight-relabeled trajectory does demonstrate how to circumvent an avoid state by wrapping around it. This sampling method is found to improve performance on the FetchReachObstacle environment (Figure 6b).

##### Sampling From a Limited Portion of the State Space.

In certain state spaces, there may be regions where sampling an avoid centroid there would not provide helpful information. Thus, instead of sampling from the entire state space, we can just sample from just the portion of the state space ${𝒮}_{\text{limited}}\subset 𝒮$ instead. This is done in the MazeObstacle and Cariogenesis environments, see Appendices C.1 and C.2 for details.

## B Model/Training Details

### B.1 Model Architectural Details and Hyperparameters

#### Representations and Embeddings for Prompt Tokens.

All prompt tokens, regardless of type, are embedded into the same latent space as the action ($a_{t}$) and state ($s_{t}$) tokens in the main trajectory sequence: ${\mathbb{R}}^{d_{h}}$, where $d_{h}$ is the hidden dimension/embedding dimension (corresponding to hyperparameter embed_dim below). **The avoid success indicator token** z is initially presented to the model as a one-hot vector: $\left[\begin{array}{l} 1\\ 0 \end{array}\right]$ if z=1 and $\left[\begin{array}{l} 0\\ 1 \end{array}\right]$ if z=0. This one-hot representation is then embedded into ${\mathbb{R}}^{d_{h}}$ via the learnable embedding matrix $E_{z}\in {\mathbb{R}}^{2\times d_{h}}$. **The avoid start token**, $i_{b}$, and **the goal start token**, $i_{g}$, are initially presented to the model as the one-hot vectors $\left[\begin{array}{l} 1\\ 0 \end{array}\right]$ and $\left[\begin{array}{l} 0\\ 1 \end{array}\right]$, respectively. They are then embedded into ${\mathbb{R}}^{d_{h}}$ via the learnable embedding matrix $E_{i}\in {\mathbb{R}}^{2\times d_{h}}$. **The prompt end start token**, e, is embedded into ${\mathbb{R}}^{d_{h}}$ as learnable vector $e\in {\mathbb{R}}^{d_{h}}$. **Avoid tokens** $b_{j}\in {\mathbb{R}}^{2d_{s}}$ and the **goal token** $g\in {\mathbb{R}}^{d_{s}}$ are embedded into ${\mathbb{R}}^{d_{h}}$ via learnable embedding matrix $E_{b}\in {\mathbb{R}}^{2d_{s}\times d_{h}}$ and learnable embedding matrix $E_{g}\in {\mathbb{R}}^{d_{s}\times d_{h}}$, respectively.

#### Attention Boosting.

We find that adding a bias adelta (a hyperparameter) to the attention logits to all prompt tokens, using the strategy described in [58], noticeably improves the instruction-following ability of RADT to the prompt—i.e., improves goal-reaching behavior without negatively impacting avoid behavior (Figure 7).

#### Model Hyperparameters.

The base causal transformer architecture we use is based on the HuggingFace implementation [61] of the GPT-2 architecture [57]; all model details that are not explicitly specified here are set to the default values for the HuggingFace implementation of GPT-2. We perform hyperparameter optimization (HPO) with a simple random search algorithm, using the RayTune framework [55]. The tunable model hyperparameters and their respective set of possible values in the search space are described in Table 3. Training hyperparameters are described in the next section.

The model hyperparameter configurations used are {n_head=4, n_layer=4, embed_dim=256, adelta=2} for the FetchReachObstacle environment, {n_head=6, n_layer=6, embed_dim=384, adelta=1} for the MazeObstacle environment, and {n_head=6, n_layer=6, embed_dim=384, adelta=1} for the Cardiogenesis environment.

**Table 3:** Model hyperparameters and search space

Hyperparameter	Description	Search space
n_head	Number of attention heads	tune.choice([1, 3, 4, 6, 12])
n_layer	Number of self-attention layers	tune.choice([3, 6, 12])
embed_dim	Size of latent embedding space (hidden dimension)	Fixed to be 64 * n_head
adelta	Attention boosting bias to the prompt	tune.choice([0, 1, 2, 3])

### B.2 Training Details

#### Loss Function.

For a batch of size B of trajectories of length T, we use the following two-component loss function during training. The first component is the typical loss used in decision transformer models, the action loss ${ℒ}_{\text{action}}$. This is the mean squared error (MSE) between the predictions of next actions $\overset{ˆ}{a_{t}}$ based on the last-layer embeddings of ($p,\tau_{1:t-1},s_{t}$) and the actual next actions $a_{t}$:

$${\left.{ℒ}_{\text{action}}=\frac{1}{B}\sum_{i=1,2,\ldots ,B}\frac{1}{T}\sum_{t=1,2,\ldots ,T}\hat{\left(a_{t}^{\left(i\right)}\right.}-a_{t}^{\left(i\right)}\right)}^{2}$$

The second component is used to encourage RADT to learn how to be aware, at each timestep, whether or not the current state $\int_{⊔}$ violates any avoid box $b_{j}\;:\;j\in \left\{1,2,\ldots ,n_{\text{avoid}}\right\}$ in prompt p. We define the indicator $k_{t}$ to indicate whether or not $s_{t}$ violates any avoid boxes $b_{j}$ in the prompt, with $k_{t}=1$ indicating that $s_{t}$ does not violate any avoid boxes and $k_{t}=0$ indicating $s_{t}$ violates at least one avoid box. During training *only*, in addition to predicting the next action $a_{t}$, we also make RADT predict $k_{t}$ using the last-layer embeddings of ($p,\tau_{1:t-1},s_{t}$). We define the box awareness loss ${ℒ}_{\text{avoid\_awareness}}$ to be the binary cross entropy (BCE) loss between predicted $\overset{ˆ}{k_{t}}$ and actual $k_{t}$.

$$\left.{ℒ}_{\text{avoid\_awareness}}=\frac{1}{B}\sum_{i=1,2,\ldots ,B}\frac{1}{T}\sum_{t=1,2,\ldots ,T}k_{t}^{\left(i\right)}log\left(\hat{k_{t}^{\left(i\right)}}\right)+\left(1-k_{t}^{\left(i\right)}\right)log\left(1-\hat{k_{t}^{\left(i\right)}}\right)\right)$$

The combined loss function is then:

$$ℒ={ℒ}_{\text{action}}+\alpha {ℒ}_{\text{avoid\_awareness}}$$

α is a tunable hyperparameter to balance the different components of the loss function. During hyperparameter optimization, we find that it does not have a significant effect on empirical performance when we let the model train for long enough, so we set it to 1 in all our experiments for simplicity.

#### Stopping Criteria.

We let all RADT models train for 50,000 training steps (no visual improvement in SR or MNC beyond that), checkpointing every 500 steps. As we use a similar training scheme as the one used in the original Prompt DT [46] and MGPO [49] papers, where we sample batches of trajectories with replacement, we cannot use “epoch” as a measurement of training progress. At every checkpointing iteration, we run an evaluation on the model in the same way we did in our experiments (described in Section 5). We choose the checkpoint with the best (lowest) MNC whose SR is within 0.05 from the checkpoint with the highest SR (to ensure we have a model that is not achieving seemingly good avoid ability by significantly sacrificing goal-reaching ability, e.g., not moving). We do the same process for all baselines, except we train the baseline for 500 epochs as is done in the RbSL paper [3] (no visual improvement in SR or MNC beyond that).

#### Training Hyperparameters.

Training hyperparameters are listed with their respective search spaces in Table 4. The scheduler value ‘lambdalr’ corresponds to the PyTorch scheduler torch.optim.lr_scheduler.LambdaLR and the scheduler value ‘cosinewarmrestarts’ corresponds to the scheduler CosineAnnealingWarmupRestarts from the cosine_annealing_warmup library [54] library is used to tune training hyperparameters. Any hyperparameters not explicitly listed default to the values used in MGPO [49].

The chosen training hyperparameter configurations are {batch_size=128, learning_rate=1e-4, scheduler=‘cosinewarmrestarts’ T_0=1000, warmup_steps=500, alpha1=1, for the FetchReachObstacle environment, {batch_size=32, learning_rate=1e-4, scheduler=‘lamdalr’, warmup_steps=1000, alpha1=1} for the MazeObstacle environment, and {batch_size=128, learning_rate=1e-4, scheduler=‘lamdalr’, warmup_steps=1000, alpha1=1} for the Cardiogenesis environment.

**Table 4:** Training hyperparameters and search space

Hyperparameter	Description	Search space
batch_size	Batch size	tune.choice([32, 64, 128, 256])
learning_rate	Maximum learning rate	tune.choice([1e-4, 1e-5])
scheduler	Learning rate scheduler	tune.choice([‘cosinewarmrestarts’, ‘lambdalr’])
warmup_steps	Number of warmup steps	tune.choice([500, 1000])
T_0	T_0 parameter to CosineAnnealingWarmupRestarts	Fixed to be 1000
T_mult	T_mult parameter to CosineAnnealingWarmupRestarts	Fixed to be 1
weight_decay	λ constant used in weight decay	Fixed to be 1e-4
dropout	Dropout ratio during training	Fixed to be 0.1
alpha1	The *α* weight balancing the two-component loss function	tune.loguniform([0.1, 10])

#### Compute Resources.

All training sessions of RADT models were done using a single H100 GPU on Harvard University Kempner Institute’s compute cluster. The longest single training session we ran for an RADT model that was used in the evaluation was 3 days (that is, 72 GPU hours).

## C Experiment Setup

### C.1 GymnasiumRobotics Environments

#### Choice of Environments.

While there are more complicated environments that are part of the Fetch and Maze suites in Gymnasium robotics, we do not evaluate on those, since the focus of those environments is testing for proficiency in long-range planning, skills learning, and hierarchical learning, which are not the focus of the current iteration of RADT. Additionally, we are focusing only on the domain where the training data is 100% generated from a random policy (Criterion 1.2), and it is difficult for RL approaches across the board to just learn good goal-reaching performance on these more complex environments under this data regime [32], let alone good avoid behavior. With low goal-reaching success rates, our evaluation of avoid behavior will not be very meaningful. As an extreme example, a policy that makes the agent stay in place will achieve an MNC of 0, but its SR will also be 0; this would not be considered good avoid behavior despite the low MNC.

#### Passable and Impassable Avoid Regions.

In our custom environments, we make the avoid regions passable (i.e., the agent can pass through these avoid states). This is in contrast to physical boxes that provide a hard constraint on the state space (i.e., the agent cannot cross the boundaries of the avoid box) as is used in [3]. Hard constraints on the state space present an issue: no training trajectories generated in this environment truly violate any $b_{j}$, because no training trajectories actually pass *through* any $b_{j}$ (Figure 3b). Thus, all trajectories are somewhat optimal, in the sense that they never demonstrate “unsuccessful” avoid behavior. This setup thus fails to demonstrate Property (2.2) in Section 2. We argue that this is an issue because it couples successful goal-reaching behavior with successful avoiding behavior. If training trajectories cannot go through avoid boxes, all training trajectories that have succeeded in reaching the goal must have done so by circumventing avoid boxes. A training trajectory where the agent is adamant on attempting to go through an avoid box will get stuck at the box boundary and fail to reach the goal. Therefore, with a hard constraint setup, goal-reaching ability is tightly coupled with avoid ability, while we wish to *isolate* these two aspects and see if a model can learn to acquire both goal-reaching and avoid abilities when it is possible to only acquire one but not the other. While this may not be important in the specific context of these Gymnasium Robotics tasks, in general, there may be application scenarios in which we would like to avoid a region of the state space that the agent is technically *able* to pass through, but we would prefer it not. For example, there may be a road in which a self-driving vehicle *can* pass through or *has* previously passed through, but we would rather it not on some particular day due to construction traffic.

#### Environment Specifications.

For the baseline models (AM-Lag, RbSL, WGCSL), the FetchReachObstacle environment state space consists of 19 dimensions. The first 10 dimensions correspond to the state space of the original FetchReach environment in Gymnasium Robotics [6] and describe information about the location/orientation of the robot arm and the environment in general. The next 9 dimensions describe information about the single obstacle/avoid region present in the environment, with the same setup as used in [3]. For RADT, the FetchReachObstacle environment just has the first 10 dimensions (i.e., the same state space as the original FetchReach environment), as RADT does not utilize an avoid-region-augmented state space. For the baseline models, the MazeObstacle environment state space consists of $4+2*n_{\text{avoid}}$ dimensions. The first 4 dimensions correspond to the state space of the original PointMaze from Gymnasium Robotics and describe the location and velocity of the agent. Then, for each avoid region in the environment, there are an additional 2 dimensions added to the state space representing the xy-location of the centroid. Thus, the state space dimensionality gets larger the more avoid regions the environment is specified to have. For RADT, however, the state space of MazeObstacle is just the state space of PointMaze and contains 4 dimensions. The avoid regions in the MazeObstacle environment are circles of radius 0.2 around the avoid centroids.

Each environment has a parameter max_episode_steps, which dictates the maximum number of timesteps in an episode of interaction with that environment. An episode ends either when the max_episode_steps number of timesteps is reached or if the agent successfully reaches the goal. max_episode_steps is set to be 50 for FetchReachObstacle and 300 for MazeObstacle, which correspond to the original default values of max_episode_steps for FetchReach and PointMaze_UMaze from Gymnasium Robotics.

#### Length-Normalized Cost Return.

We observe in the visualizations of our preliminary experiments that it is possible to “hack” absolute cost return by attempting to reach the goal state in as few timesteps as possible and rushing directly through the avoid region (Figure 8). Such a policy, which we shall refer to as the “rushed policy,” can achieve low absolute cost by minimizing the number of timesteps in the trajectory in total, and thus also minimizing the absolute number of timesteps spent in the avoid region. However, for reach-avoid applications where safety can be critical, the rushed policy is not preferred to a slower, more cautious policy (which we shall refer to as the “cautious policy”) that may take more timesteps to reach the goal but demonstrates a stronger attempt at circumventing the avoid region. The cautious policy may result in a trajectory that accumulates a similar absolute cost return as the rushed policy trajectory (because it may have skimmed the avoid region for a similar absolute number of timesteps as the rushed policy spent in the avoid region), but because the overall cautious policy trajectory makes a better attempt at circumventing the avoid region, it achieves a much lower length-normalized cost return compared to the rushed policy trajectory. That is, a smaller proportion of the timesteps in the cautious policy trajectory violate the avoid region compared to the rushed policy trajectory, indicating higher quality avoid behavior compared to the rushed policy trajectory. In safety-critical contexts, as long as the agent can reach the desired goal in a reasonable time (i.e., the specified max_episode_steps), the *quality* of avoid behavior is more important than the speed of reaching the goal. Figure 8 demonstrates this with two visual examples.

#### Avoid Region Relabeling.

For training data from the FetchReachObstacle environment, hindsight-relabeled avoid centroids are sampled according to the contour-based sampling method described in A.2 For training data from the MazeObstacle environment, hindsight-relabeled avoid centroids are sampled using the naive uniform strategy, but only from the parts of the state space that are accessible by the agent; i.e., avoid centroids cannot be sampled inside the walls of the mazes. This is to prevent the model from conflating dynamically specified avoid regions in the prompt with hard constraints on the state space that are inherent to the environment. Since the avoid regions in MazeObstacle are circles of radius 0.2, we choose the circumscribing box of width 0.4 around the avoid centroid for our avoid box representation in RADT, a conservative choice.

#### Baselines Cannot Generalize Zero-shot.

Here, we clarify in more concrete examples why zero-shot generalization to avoid box sizes and numbers is not feasible with AM-Lag and RbSL as they are set up in [3].

For the FetchReachObstacle tasks, assume at training time the avoid boxes b in the environment have width w. The augmented state space only includes information about the center of the avoid box: centroid(*b*). The *size* of the avoid box is encoded by the cost function. The cost function that AM-Lag and RbSL are trained on outputs a cost of 1 if the agent’s state is within the box of width w surrounding centroid(*b*), otherwise, it outputs 0. Now, say we increase the avoid box sizes to 2w at evaluation time. The only information the agents get about the avoid region at evaluation time is in the centroid information centroid(*b*) present in the state space, which carries no information about the box size. The agents are still trained to stray away from the box of width w surrounding centroid(*b*), as this is the cost function they are trained to minimize the value of. Thus, to generalize to this new box size, we must define a new cost function that outputs a cost of 1 if the agent’s state is within the box of width 2w surrounding centroid(*b*), then *retrain* the agents on this new cost function.

For the MazeObstacle tasks, assume at training time that there are 3 avoid regions $b_{1},b_{2},b_{3}$ in the environment. The augmented state space for AM-Lag and RbSL at training time thus has dimensionality 4 + 2 + 2 + 2 = 10, with 2 additional dimensions for each of centroid$\left(b_{1}\right)$, centroid$\left(b_{1}\right)$, centroid$\left(b_{1}\right)$ (see Environment Specifications above). Say, at evaluation time, we increase the number of avoid regions to $5:b_{1},b_{2},b_{3},b_{4},b_{5}$. Now the state space dimensionality is 4+2+2+2+2+2=14. We will have to retrain the agents on this new state space.

### C.2 Cardiogenesis Environment

#### Cardiogenesis Boolean Network Details.

The boolean network model consists of 15 genes (nodes) and is depicted by Figure 9a and described mathematically in [36, 15]. Updates to the boolean network are done asynchronously, as is done in [36]: a random gene node is chosen, and its value is set (either changed or remained in place) such that it satisfies all boolean rules. A *stable attractor state* is defined as a state in which all boolean rules in the boolean network are satisfied, such that the boolean network is self-consistent and further asynchronous updates will not change the node values (assuming absence of an external perturbation).

In our Cardiogenesis environment simulations, a state-action-state transition sequence is obtained as follows: 1) we start with the boolean network representing the current gene expression state $s_{t}$, 2) an action is chosen (i.e., a chosen gene node is perturbed and has its value flipped from 0 to 1 or vice versa) to create a post-perturbation *transient state*, 3) k asynchronous updates to the boolean network are performed to the network, and 4) the resulting state after k asynchronous updates is defined to be the next state in the trajectory, $s_{t+1}$. This is depicted in Figure 9b. Since the boolean network updates are asynchronous, there is some stochasticity involved in the transitions due to the random selection of genes to be updated at each asynchronous update step. The value of k affects how stochastic our transitions are; the higher the value of k, the more likely the boolean network model will hit a *stable attractor state* in the process, and the less noisy the transitions of our Cardiogenesis environment will be. We choose k=10. Note that this value of k does not guarantee that all states $s_{t}$ will be stable attractor states; this is intentional, since in reality, a cell can be perturbed while it is unstable and still in the process of reaching a stable attractor state.

#### Avoid Region Relabeling.

At training time, the offline dataset of 60,000 random policy steps is split into trajectories of length 30. Hindsight goal relabeling is done as usual. During hindsight avoid-region relabeling, we sample avoid regions from the top 20 most represented states (attractors and non-attractors) in the offline dataset. This choice results in approximately half of all training trajectories being successful demonstrations of avoid behavior on the first pass of hindsight avoid-region relabeling. Since we are working with a discrete state space, avoid states that are discrete states rather than regions of a continuous state space. However, given an avoid state vector $\left[\begin{array}{llll} b_{1} & b_{2} & \ldots & b_{15} \end{array}\right]$ : $b_{i}\in \{0, 1\}$, we can still create an avoid box representation $b\in {\mathbb{R}}^{30}$ by adding some small, arbitrary margin ϵ around each dimension to create a box:

$$b=\left[\begin{array}{lllllll} \left(b_{1}-\epsilon \right) & \ldots & \left(b_{15}-\epsilon \right) & | & \left(b_{1}+\epsilon \right) & \ldots & \left(b_{15}+\epsilon \right) \end{array}\right]$$

We choose ϵ=0.001 arbitrarily.

#### Start State/Goal State Sampling.

At evaluation time, start states are randomly chosen from stable attractor states. The goal state is fixed as the FHF state (000010010100000) for our experiment.

#### Example Trajectories from Experiments.

Here, we show some example trajectories depicting the three scenarios described in Section 5.3. Note that, unless otherwise specified, we depict trajectories where repeated states are collapsed, which we call the “collapsed trajectory.” For example, the trajectory (a, a, b, c, d, d, d) would be collapsed into the collapsed trajectory (a, b, c, d). It is important to note that it is possible for an action to lead to staying in the same state due to the dynamics of the Boolean network model.

As an example of Scenario 1, when the starting state is 000000000000000, RADT’s policy, when given no avoid prompt, always leads to a collapsed trajectory (000000000000000, 100000000001100, FHF). However, upon adding 100000000001100 as an avoid prompt, the resulting policy always leads to a collapsed trajectory (000000000000000, 000011010100000, 000010010100000, FHF), which is a longer, alternative path that deterministically circumvents 100000000001100.

In Scenario 2, the stochastic environment plays a much more prominent role. For example, when the starting state is 010111111010011, and RADT is given no avoid prompt, the resulting policy leads to a variety of trajectories. A few examples are: (010111111010011, 001111011011111, FHF), (10111111010011, 010111111010011, 001111011011111, 000111010011111, 001111011011111, 100000000001100, FHF), and (010111111010011, 001111011011111, 000111010011111, 001111011011111, FHF). Upon adding 001111011011111 as an avoid prompt, we still get some similar trajectories involving 001111011011111 as an intermediate state, but we get a higher frequency of trajectories that manage to avoid it, such as (010111111010011, 000000000000011, 010010010100000, 010111111010011, 000010010100000, FHF) and (010111111010011, 000010010100000, 010111111010011, 000010010000000, FHF). The takeaway here is the diversity of trajectories that are possible given the same policy due to the stochastic nature of the environment.

As an example of Scenario 3, when the starting state is 000010010100000, all trajectories have collapsed form (000010010100000, FHF) when there is no avoid prompt, meaning the cell can directly reach the goal state from the start state without passing through a third intermediate state. However, the uncollapsed form of these trajectories has length 5 and is (000010010100000, 000010010100000, 000010010100000, 000010010100000, 000010010100000, FHF), indicating that RADT makes multiple attempts to get out of 000010010100000 to land on FHF and only manages to get out on the 5th attempt, spending a total of 5 timesteps stuck in the initial state 000010010100000. However, when we add the initial state 000010010100000 as an avoid prompt (which is unavoidable since it is the starting state), the uncollapsed form of all resulting trajectories has length 3 instead and is (000010010100000, 000010010100000, 000010010100000, FHF). Here, RADT cannot avoid the avoid state 000010010100000 entirely, but it is encouraged and finds a way to spend less time at the 000010010100000 state, getting out of the state more quickly (40% fewer attempts).

### C.3 Other Notes

#### Lidar-based Observation Spaces and Goal-Conditioning.

Regarding the discussion about OSRL approaches in Section 3, it is worth noting that the standard “reach avoid” benchmarking task for OSRL algorithms, the Safe Navigation environment from Safety Gymnasium [35, 13], may seem like it is testing the goal-conditioning and avoid region-conditioning abilities of OSRL models, but it does not require true goal-conditioning. This is because it utilizes a relative-perspective, lidar-based observation space ${𝒮}_{\text{lidar}}$ that allows the agent to only have to learn to reach *one* state in ${𝒮}_{\text{lidar}}$ in order to “generalize” to any target location in physical space: the 0 vector (indicating that the agent is 0 distance away from the goal). Therefore, models do not need to generalize to any arbitrary goal in the observation space ${𝒮}_{\text{lidar}}$, and thus OSRL models do not need to be truly goal-conditioned in ${𝒮}_{\text{lidar}}$ (most are not). The same logic can be applied to argue that the tasks do not check for true avoid region-conditioning in the observation space. While relative-perspective observation spaces are advantageous in this regard, they cannot be conceived or used in all environments, e.g., cell reprogramming.

## D Code

The code for this project will be released as a GitHub repository.

## E Detailed Related Work

Below are some additional discussions regarding the categories of related work described in Section 3 and their limitations with regard to satisfying the ideal properties described in Section 2

### Offline Goal-Conditioned RL.

While reward-based OGCRL can technically satisfy Property (4) by designing a reward function that takes into account both goal information and avoid region information (e.g. positive reward for approaching the goal, negative reward for approaching an avoid region), they *effectively* fails to satisfy Property (4) in practice. While it is theoretically possible to express information about both desirable and undesirable states in a multi-component reward function, such functions are practically difficult to design such that both these sometimes conflicting desires are balanced properly [18, 19, 9]. Also, the reason why reward-free OGCRL algorithms strictly do not satisfy Property (4) is because, without a reward, there is no way to specify any additional desires outside of the goal g, such as avoid regions. Thus, reward-free OGCRL is usually a good fit in situations where the path to the goal does not matter or the demonstrations used for training are expert/optimal demonstrations that take an ideal path to the goal.

### Offline Safe RL.

OSRL approaches circumvent the challenge of having to design multi-component reward functions by using a separate cost function to capture information about avoid behavior and solving a constrained Markov decision process [52], isolating the handling of goal-reaching and avoiding desires. We claim in Section 3 that OSRL algorithms are not truly goal-conditioned in general; however, this may seem surprising since a very common benchmark for OSRL algorithms is a reach-avoid environment called Safe Navigation from Safety Gymnasium [35]. However, we explain in Appendix C.3 why the tasks in this environment are not true multi-goal RL tasks.

### *Online* Goal-Conditioned Safe RL.

While the RbSL paper [3] (and the associated algorithms RbSL and AM-Lag) is one of the only pieces of literature we have found that explicitly attempts to create an approach that is *offline*, goal-conditioned, and avoids region-conditioned, we acknowledge that there exist other goal-conditioned, avoid region-conditioned algorithms designed for reach-avoid tasks that are *online* algorithms [8, 37, 16]. Online algorithms may work in domains like robotics, where we can reasonably create safe testing environments or have good simulators, but we would like to create an approach for domains in which online training is not feasible.

### Decision Transformers.

We build our model off of MGPO, which is truly goal-conditioned and thus satisfies Property (3). During the offline pretraining phase, MGPO is purely data-driven and does not use a reward function, fully relying on hindsight goal relabeling. However, MGPO does use an online prompt optimization phase in addition to offline pretraining (failing to satisfy Properties (1) and (2)), utilizes reward functions during online finetuning (ultimately failing to satisfy Properties (5)), and does not explicitly take into account avoid region information, unless it is baked into the reward function design (effectively failing to satisfy Properties (4)). Our work builds upon MGPO such that it is more ideal for reach-avoid tasks, aiming to satisfy these remaining properties.

## G Additional References

### Illustration Citations (NIAID NIH Bioart)

[The following images are included in our figures.]

NIAID Visual & Medical Arts., (10/7/2024). Fibroblast. NIAID NIH BIOART Source. bioart.niaid.nih.gov/bioart/152,a.

NIAID Visual & Medical Arts., (10/7/2024). Fibroblast. NIAID NIH BIOART Source. bioart.niaid.nih.gov/bioart/153,b.

NIAID Visual & Medical Arts., (10/7/2024). Fibroblast. NIAID NIH BIOART Source. bioart.niaid.nih.gov/bioart/154,c.

NIAID Visual & Medical Arts. (10/7/2024). Generic Im- mune Cell. NIAID NIH BIOART Source. bioart.niaid.nih.gov/bioart/173.

NIAID Visual & Medical Arts. (10/7/2024). Human Male Outline. NIAID NIH BIOART Source. bioart.niaid.nih.gov/bioart/232.

NIAID Visual & Medical Arts. (10/7/2024). Intermediate Progenitor Cell. NIAID NIH BIOART Source. bioart.niaid.nih.gov/bioart/258
